# Carbon Monoxide Abrogates Ischemic Insult to Neuronal Cells via the Soluble Guanylate Cyclase-cGMP Pathway

**DOI:** 10.1371/journal.pone.0060672

**Published:** 2013-04-08

**Authors:** Nils Schallner, Carlos C. Romão, Julia Biermann, Wolf A. Lagrèze, Leo E. Otterbein, Hartmut Buerkle, Torsten Loop, Ulrich Goebel

**Affiliations:** 1 Department of Anesthesiology, Division for Experimental Anesthesiology, University Medical Center Freiburg, Germany; 2 Instituto de Tecnologia Química e Biológica, Universidade Nova de Lisboa, Oeiras, Portugal; 3 University Eye Hospital, University Medical Center Freiburg, Germany; 4 Beth Israel Deaconess Medical Center, Department of Surgery, Harvard Medical School, Boston, Massachusetts, United States of America; 5 Alfama Limitida, Instituto de Biologia Experimental e Tecnológica, Oeiras, Portugal; University of Oldenburg, Germany

## Abstract

**Purpose:**

Carbon monoxide (CO) is an accepted cytoprotective molecule. The extent and mechanisms of protection in neuronal systems have not been well studied. We hypothesized that delivery of CO via a novel releasing molecule (CORM) would impart neuroprotection *in vivo* against ischemia-reperfusion injury (IRI)-induced apoptosis of retinal ganglion cells (RGC) and *in vitro* of neuronal SH-SY5Y-cells via activation of soluble guanylate-cyclase (sGC).

**Methods:**

To mimic ischemic respiratory arrest, SH-SY5Y-cells were incubated with rotenone (100 nmol/L, 4 h) ± CORM ALF186 (10–100 µmol/L) or inactivated ALF186 lacking the potential of releasing CO. Apoptosis and reactive oxygen species (ROS) production were analyzed using flow-cytometry (Annexin V, mitochondrial membrane potential, CM-H_2_DCFDA) and Western blot (Caspase-3). The impact of ALF186± respiratory arrest on cell signaling was assessed by measuring expression of nitric oxide synthase (NOS) and soluble guanylate-cyclase (sGC) and by analyzing cellular cGMP levels. The effect of ALF186 (10 mg/kg iv) on retinal IRI in Sprague-Dawley rats was assessed by measuring densities of fluorogold-labeled RGC after IRI and by analysis of apoptosis-related genes in retinal tissue.

**Results:**

ALF186 but not inactivated ALF186 inhibited rotenone-induced apoptosis (Annexin V positive cells: 25±2% rotenone vs. 14±1% ALF186+rotenone, p<0.001; relative mitochondrial membrane potential: 17±4% rotenone vs. 55±3% ALF186+rotenone, p<0.05). ALF186 increased cellular cGMP levels (33±5 nmol/L vs. 23±3 nmol/L; p<0.05) and sGC expression. sGC-inhibition attenuated ALF186-mediated protection (relative mitochondrial membrane potential: 55±3% ALF186+rotenone vs. 20±1% ODQ+ALF186+rotenone, p<0.05). ALF186 protected RGC *in vivo* (IRI 1255±327 RGC/mm^2^ vs. ALF186+IRI 2036±83; p<0.05) while sGC inhibition abolished the protective effects of ALF186 (ALF186+IRI 2036±83 RGC/mm^2^ vs. NS-2028+ALF186+IRI 1263±170, p<0.05).

**Conclusions:**

The CORM ALF186 inhibits IRI-induced neuronal cell death via activation of sGC and may be a useful treatment option for acute ischemic insults to the retina and the brain.

## Introduction

Ischemic injury to neuronal cells might occur in the perioperative period during major cardiovascular but also non-cardiac surgical procedures [1]. Against current dogma that carbon monoxide (CO) is poisonous, particularly to the brain, emerging data suggest that at low doses CO provides potent neuroprotection by its anti-inflammatory and anti-apoptotic properties [2], [3]. The application of CO releasing molecules (CORMs) may be a valuable alternative to inhaled CO, because they can be administered to biological systems via systemic routes and potentially in a tissue-specific manner thus avoiding unreliable inhaled application and allowing safe administration to target organs such as the central nervous system. CORMs suppress the inflammatory response in glial cells *in vitro* [4] and might therefore exhibit neuroprotection. However, other findings have elicited some confusion as to the consistency of the neuroprotective effects, which may reflect the choice of CORM. The well-described ruthenium based, water-soluble CORM-3 is effective when given before or late after neuronal injury but not when given early afterwards [5]. Here, we present data that a molybdenum-based, water-soluble CORM (ALF186) possesses a different CO release profile releasing CO in a dose- and oxygen-dependent manner after administration [6]. ALF186 has shown protective effects *in vivo* in models of acute inflammation [7], [8] and in the regulation of vasomotor tone [9].

Given the anti-inflammatory and vascular effects of CO and ALF186 we hypothesized that ALF186 would be an ideal modality to assess the potential neuroprotective properties of ALF186 *in vitro* and *in vivo* in models of ischemic injury. The mechanisms of CORM-mediated protective effects have not been well studied in neuronal systems. This is of particular importance considering the large amount of data that CO may be neurotoxic. Heme-containing proteins are well-described cellular targets given the affinity of CO for ferrous iron in heme. Binding of CO to heme can alter enzymatic function both positively or negatively and include soluble guanylate-cyclase (sGC), nitric oxide synthase (NOS) and NADPH oxidase as well as the mitochondrial cytochrome c oxidase complexes [10]. The role of these “primary targets” in mediating anti-inflammatory [11], anti-proliferative [12] and vasoactive [13] effects of CORMs have been characterized in non-neuronal systems, but not in neuronal systems. Indirect effects of CO on non-heme proteins have also been shown to contribute to its protective effects [10].

CO has emerged as a gaseous neurotransmitter that modulates neuronal cGMP levels [14]. CO and CORMs are known sGC-activators, leading to a moderate increase in cGMP production [9], [13], [15]. Growing evidence supports a role for the sGC-cGMP pathway in CO-mediated protection of neuronal cells against apoptosis [2], [16]. However, little is known whether CORM-mediated neuroprotective effects occur through this pathway. Protective effects that occur through activation of sGC-cGMP signal transduction seem to be mediated through protein kinase G1 (PKG1) activation [16] albeit the distinct cellular downstream targets for these protective effects remain unknown.

## Materials and Methods

### Reagents

**Figure 1:**
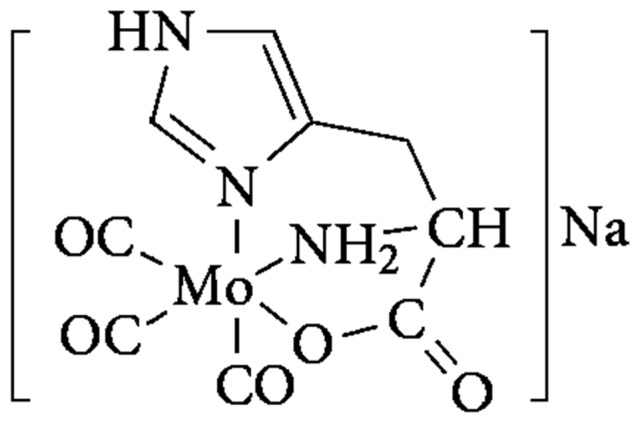
Chemical structure of ALF186. Carbon monoxide liberating compound containing a central molybdenum atom.

The carbon monoxide releasing molecule ALF186 ([Mo(CO)_3_(histidinato)]Na; **Fig. 1** **;** Alfama Inc., Lisbon, Portugal) was kindly provided by C. Romão [6], [8]. It was freshly dissolved in PBS prior to cell culture treatment. The inactive compound iALF186 was prepared by dissolving ALF186 in PBS and incubated for 24 h under exposure to air and light. After 24 h, the solution was bubbled with nitrogen to remove residual carbon monoxide.

Rotenone was purchased from Sigma Aldrich (Taufkirchen, Germany). It was freshly prepared by dissolving in dimethylsulfoxide (DMSO) prior to the experiments. DMSO concentration in cell culture media did not exceed 0.5%.

The inhibitors of proteinkinase G-1(PKG1; KT5832), the inhibitor (ODQ) and the inductor (YC-1) of the soluble guanylate-cyclase (sGC) and the cGMP-analogue 8-Br-cGMP were obtained from Sigma Aldrich, as were the inhibitors of the nitric oxide synthase (NOS) L-NAME and of the NADPH oxidase DPI (diphenylene iodonium).

### Cell Culture and Treatment

Neuroblastoma cells (cell line SH-SY5Y; ATCC No. CRL-2266) were cultured in DMEM medium (with penicillin/streptomycin, 1% glutamate and 10% fetal bovine serum) in a humidified atmosphere with 5% carbon dioxide at 37°C constant temperature until 80% confluence was achieved. The cells were usually seeded in 6 well culture plates at a density of approximately 3×10^5^ per well 24 h prior to individual treatment. Before rotenone treatment, the cells were transferred into media containing 0.5% fetal bovine serum, to prevent inactivation of rotenone by protein binding. Cells were pre-treated with 10–100 µmol/L ALF186 or iALF186 30 min before induction of apoptosis with 100 nM Rotenone. After 4 h of rotenone-treatment, cells were collected for quantification of apoptosis or protein and gene expression analysis. For assays analyzing the influence of ALF186 alone without rotenone-treatment, cells were collected 1 h after ALF186 treatment.

### Quantification of Apoptosis

Staining with Annexin V-FITC and propidiumiodide (Becton Dickinson, Heidelberg, Germany) and flow-cytometric analyses were done as previously described [17]. Western Blots were done as previously described [17] using antibodies against cleaved Caspase-3 (#9664 from Cell Signaling Technology, Danvers, MA, USA) and GAPDH (#CSA-335 from Enzo Lifesciences, Plymouth, PA, USA).

### Quantification of Mitochondrial Membrane Potential Loss

Mitochondrial membrane potential (ΔΨ_m_) was quantified using the Mito-Probe JC-1 Assay (Molecular Probes, Invitrogen, Darmstadt, Germany). SH-SY5Y cells were stained with 2 µmol/L JC-1 for 15 min prior to cell collection after treatment with rotenone, ALF186 or inhibitors as indicated in the individual experiments. Cells were analyzed using flow-cytometry. JC-1 exhibits mitochondrial membrane potential-dependent accumulation in mitochondria, which leads to formation of aggregates and a fluorescence emission shift from green to red. As a consequence, mitochondrial depolarization is indicated by a decrease in the ratio of red/green fluorescence intensity. The ratio between red and green fluorescence intensity was calculated. While the ratio in untreated cells was set to 100%, the difference compared to treated cells was used to describe a loss in mitochondrial membrane potential.

### Analysis of Primary Carbon Monoxide Targets

Soluble guanylate cyclase (sGC) and corresponding cGMP were analyzed using a cGMP detection kit (Cell Signaling Technology, Danvers, MA, USA). Expression of the sGC α and β_1_ subunits were quantified using RT-PCR, while the subunit proteins were visualized with Western blot analyses (sGC α_1_: #160895; sGC β_1_: #160897 from Cayman Chemical, Ann Arbor, MI, USA). Selective induction of sGC was achieved using YC-1 and selective inhibition via ODQ using 20 µmol/L for 30 min and 10 µmol/L for 30 min respectively. Also, a cell permeable cGMP-analogue (8-Br-cGMP, 1 mmol/L, 30 min) was used to mimic sGC activation. Protein kinase G-1 (PKG1, cGK1) as a downstream target of cGMP was inhibited with the selective inhibitor KT5823 (1 µmol/L, 1 h). Expression of the inducible (i) and neuronal (n) isoforms of nitric oxide synthase (NOS) was analyzed using Western blot (iNOS: #2977; nNOS: #4236 from Cell Signaling Technology, Danvers, MA, USA). Inhibition of NOS was achieved by incubation with L-NAME (50 µmol/L, 30 min) and inhibition of NADPH oxidase was performed using diphenylene iodonium (DPI, 10 µmol/L, 1 h).

### Quantification of Nitric Oxide Production

Nitric oxide production was quantified by measurement of total nitrite concentration in cell supernatants using the Griess Method (Nitric Oxide Colorimetric Assay Kit, BioVision, Milpitas, CA, USA) following the manufacturer’s instructions. Absorbance at 540 nm was then read on a microplate reader (Molecular Devices, SpectraMax, Biberach, Germany) to quantify total nitrite content in the samples.

### Reactive Oxygen Species

Quantification of reactive oxygen species (ROS) was assessed using the fluorescence dye CM-H_2_DCFDA (Invitrogen, Karlsbad, Germany) by flow-cytometry. Incorporation of CM-H_2_DCFDA is indicative of intracellular formation of ROS and can be assessed by quantification of fluorescence intensity in FL-1. A positive control was obtained using phorbol-myristate-acetate (PMA, Sigma Aldrich, Taufkirchen, Germany) at a concentration of 1 µmol/L for 5 min.

### Animals

Adult male and female Sprague-Dawley rats (1∶1, 280–350 g body weight, Charles River, Sulzfeld, Germany) were used. Animals were fed a standard rodent diet *ad libitum* while kept on a 12-h light/12-h dark cycle. All procedures involving animals concurred with the approval of The Association for Research in Vision and Ophthalmology and were approved by the Committee of Animal Care of the University of Freiburg (Permit Number: 35-9185.81/G-11/81). All types of surgery and manipulations were performed under general anesthesia with isoflurane/O_2_ for retrograde labeling with fluorogold or a mixture of intraperitoneally administered ketamine 50 mg/kg (Ceva-Sanofi, Germany) and xylazine 2 mg/kg (Ceva-Sanofi) for the ischemia reperfusion experiments. Body temperature was maintained at 37±0.5°C with a heating pad controlled by a rectal thermometer probe. After surgery, buprenorphine (50 µg/kg; Essex Pharma, Germany) was applied subcutaneously to treat pain. While recovering from anesthesia, animals were placed in separate cages and gentamicin ointment (Refobacin®; Merck, Darmstadt, Germany) was applied on ocular surfaces and skin wounds. The number of animals used for RGC quantification and molecular analysis was n = 6 per group.

### Retrograde Labeling of RGC

Rats were anesthetized with isoflurane, placed in a stereotactic apparatus (Stoelting, Kiel, Germany) and retrograde RGC-labeling was done as described previously [18]: The skin overlying the skull was cut open und retracted. The lambda and bregma sutures served as landmarks for drilling 3 holes on each site of the bregma suture. A total amount of 7.8 µl fluorogold (FG) (Fluorochrome, Denver, CO, USA) dissolved in 10% dimethylsulfoxide in PBS was injected into both lateral superior colliculi through the drilling holes. To ensure adequate retrograde transport of FG into the RGC’s perikarya, further experimental interventions were done 7 days after retrograde labeling.

### Retinal Ischemia/Reperfusion Injury and Treatment with ALF186

Rats were anesthetized intraperitoneally with xylazine and ketamine. To evaluate a neuroprotective effect of carbon monoxide released from ALF186, animals were randomized to receive treatment with ALF186 (10 mg/kg body weight i.v., dissolved in PBS) or PBS (vehicle controls) alone 30 min prior to initiation of ischemia. A third group received NS-2028 (inhibitor of sGC; Sigma Aldrich; 7 mg/kg body weight i.v. dissolved in DMSO/PBS) 30 min before ALF186 treatment and subsequent ischemia/reperfusion injury. Retinal ischemia/reperfusion injury was performed as described previously [3]. Briefly the anterior chamber of the left eye was cannulated with a 30-gauge needle connected to a reservoir containing 0.9% NaCl. Intraocular pressure was increased to 120 mm Hg for 60 min and ocular ischemia was confirmed microscopically by interruption of the ocular circulation. Rats without immediate recovery of retinal perfusion at the end of the ischemic period or those with lens injuries were excluded from the investigation, since the latter prevents RGC death and promotes axonal regeneration.

### RGC Quantification

Animals were killed by CO_2_-inhalation 7 days after ischemia. Retinal tissue was immediately harvested, placed in ice-cold Hank´s balanced salt solution and further processed for whole mount preparation. Retinae were carefully placed on a nitrocellulose membrane with the ganglion cell layer (GCL) on top. After removing the vitreous body, retinae were fixed in 4% paraformaldehyde for 1 h and then embedded in mounting media (Vectashield; Axxora, Loerrach, Germany). The densities of FG-positive RGC were determined using a fluorescence microscope (AxioImager; Carl Zeiss, Jena, Germany) and the appropriate bandpass emission filter (FG: excitation/emission, 331/418 nm), as previously described [19]. Briefly, we photographed 3 standard rectangular areas (0.200 mm×0.200 mm = 0.04 mm^2^) at 1, 2 and 3 mm distal from the optic disc in the central regions of each retinal quadrant. Hence, we evaluated an area of 0.48 mm^2^ per retina (12×0.04 mm^2^). Assuming an average retinal area of about 50 mm^2^ in rats [20], we evaluated about 1% of the retina. To calculate the average RGC density in cells/mm^2^, we multiplied the number of analyzed cells/0.04 mm^2^ by 25. Secondary fluorogold stained activated microglia cells (AMC) after RGC phagocytosis were identified by morphologic criteria and excluded from calculation. All averaged data are presented as mean RGC densities [cells/mm^2^] ± SD.

### Real Time Polymerase Chain Reaction (RT-PCR)

For the *in vivo* experiments, retinal tissue was harvested 12 h after IRI. For the *in vitro* experiments with SH-SY5Y neuroblastoma cells, cells were lysed 1 h after ALF186 or 4 h after rotenone treatment. Total RNA from each retina or from 0.5×10^6^ SH-SY5Y cells was extracted using a column-purification based kit (RNeasy Micro Kit®, Qiagen, Hilden, Germany) according to manufacturer’s instructions. Reverse transcription was performed with 25 to 250 ng of total RNA using random primers (High Capacity cDNA Reverse Transcription Kit, Applied Biosystems, Darmstadt, Germany). RT-PCR was done with a TaqMan® probe-based detection kit (Applied Biosystems), using the following primers (all from Applied Biosystems; Gene of interest/Assay ID): 1. sGC α_1_ human/Hs01015570_m1; 2. sGC β_1_ human/Hs00168336_m1; 3. GAPDH human/4326317E; 4. Bax rat/Rn02532082_g1; 5. Bcl-2 rat/Rn99999125_m1; 6. Caspase-3 rat/Rn00563902_m1; 7. sGC α_1_ rat/Rn00567252_m1; 8. sGC β_1_ rat/Rn00562775_m1; 9. GAPDH rat/4352338E.

PCR assays were then performed on a RT-PCR System (ABI Prism 7000, Applied Biosystems) with the following cycling conditions: 95°C for 10 min, 40 cycles of 95°C for 10 sec and 60°C for 1 min. Reaction specificity was confirmed by running appropriate negative controls. Cycle threshold (CT) values for each gene of interest were normalized to the corresponding CT values for GAPDH (ΔCT). Relative gene expression in IR-injured retinal tissue was calculated in relation to the corresponding gene expression in the non-injured retinal tissue of each individual animal (ΔΔCT). For *in vitro* experiments, relative gene expression in cells treated with ALF186 and rotenone was calculated in relation to gene expression in untreated cells (ΔΔCT).

### Statistical Analyses

All data with normal distribution are presented as means ± SD. Data without normal distribution are presented as median ±25^th^/75^th^ percentile. For *in vitro* studies, one-way ANOVA was used for between-group comparisons with post hoc Holm Sidak test and Kruskal Wallis one-way ANOVA on ranks with post hoc SNK-test for data with lack of normal distribution. For the *in vivo* studies, we wished to detect a 50% difference in RGC densities by intervention with ALF186. Based on previously published data [3] and assuming an expected SD of 10%, an a priori power analysis (α = 0.05 with two-sided hypothesis, power 80%) indicated that a sample size of six animals per group would be sufficient to detect such a difference. Two-way ANOVA (RGC densities: Factor A = ischemia with two levels: 1. control and 2. IRI; factor B = intervention with three levels: 1. vehicle, 2. ALF186, 3. NS-2028+ALF186) was used for between-group comparisons with post hoc Holm-Sidak test. Data were analyzed with a computerized statistical program (SigmaStat for Windows Version 3.1, Systat Software Inc., San Jose, CA, USA). *P*<0.05 was considered statistically significant.

## Results

### ALF186 Attenuates Rotenone-induced Apoptosis

**Figure 2:**
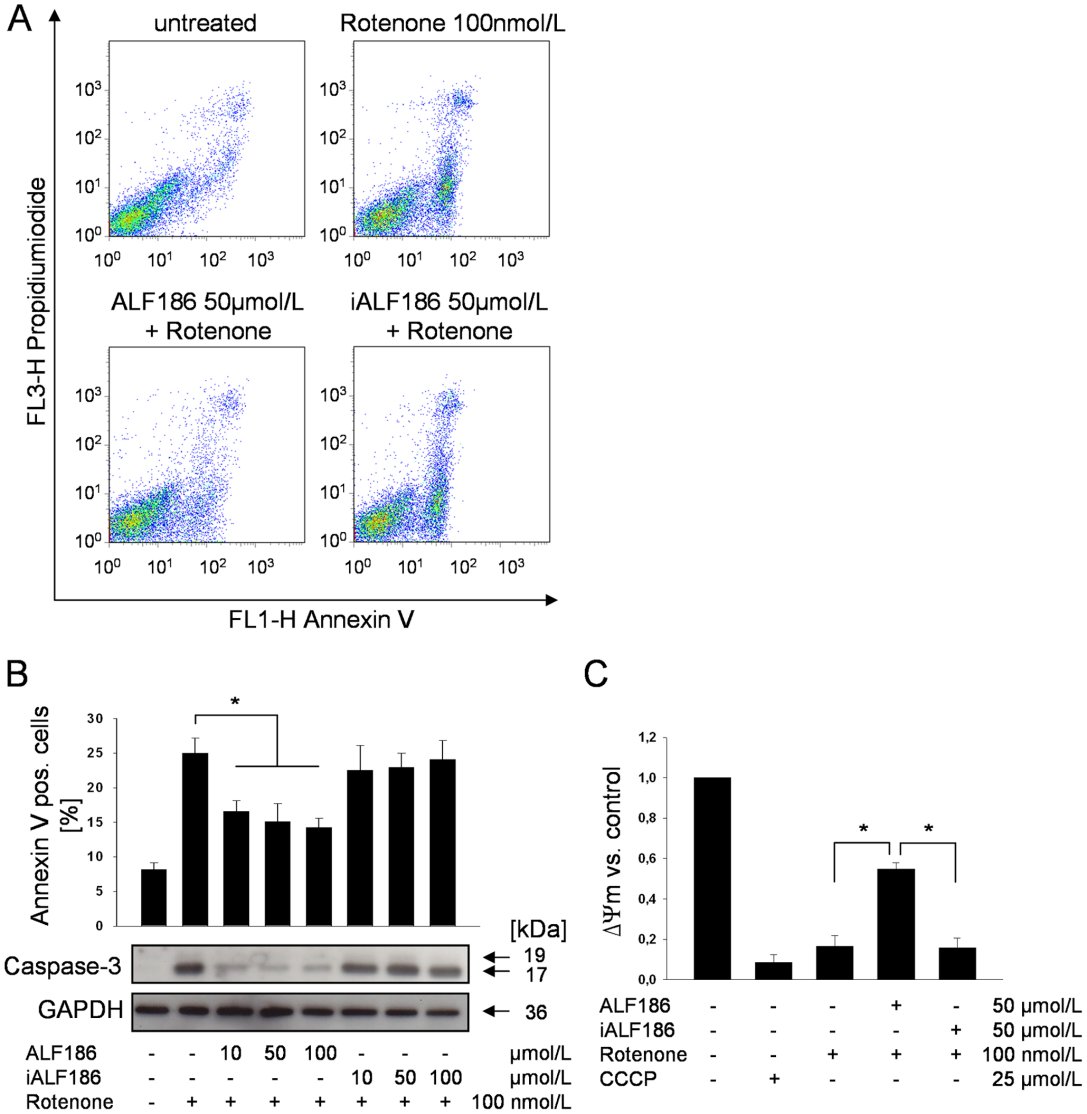
Effect of ALF186 on rotenone-induced apoptosis. **A:** Representative experiment after FITC Annexin V and propidiumiodide staining and flow-cytometric analysis. 1×10^4^ cells in each experiment were analyzed. **B:** ALF186-mediated effect on apoptosis analyzed by Annexin V staining (top, n = 6; mean±SD; * = p<0.001 rotenone vs. ALF186 10, 50 and 100 µmol/L+rotenone) and caspase-3 cleavage (bottom, representative Western blot of caspase-3 cleavage products). **C:** Flow-cytometric analysis of mitochondrial membrane potential change relative to untreated cells (ΔΨ_m_, n = 6; mean±SD; * = p<0.05 rotenone vs. ALF186+ rotenone and ALF186+ rotenone vs. iALF186+ rotenone).

The ability of CO to prevent apoptosis of non-neuronal cells has been well described and thus led us to investigate the neuroprotective properties of ALF186 on cell death. We induced apoptosis with the mitochondrial poison rotenone in SH-SY5Y neuroblastoma cells in order to mimic the changes in cellular respiration *in vitro* as occurs during IRI *in vivo*. Exposure of cells to rotenone significantly increased the percentage of Annexin V positive cells (**Fig. 2A–2B**). ALF186, but not inactivated ALF186 (iALF186) decreased the number of Annexin V positive SH-SY5Y cells (rotenone-treated 25±2% vs. ALF186 10 µmol/L+rotenone 17±2%, ALF186 50 µmol/L +rotenone 15±2%, ALF 100 µmol/L+rotenone 14±1%, all p<0.001) indicating that CO was likely mediating the neuroprotection (**Fig. 2A–2B**). To further corroborate the Annexin findings we measured cleavage of caspase-3 as a marker of apoptosis. Rotenone caused the degradation of pro-caspase-3 and formation of caspase-3 cleavage products (**Fig. 2B**), which was abrogated in the presence of ALF186. Importantly, the inactive compound without CO (iALF186) had no effect on caspase-3 cleavage.

Since rotenone inhibits the mitochondrial electron transfer chain at complex I, we next analyzed the influence of ALF186 on rotenone-induced mitochondrial depolarization as an indicator of apoptosis. Rotenone induced an 87% decrease in mitochondrial membrane potential over naïve controls (p<0.05), which was comparable to depolarization after treatment with cyanide (8±4% mitochondrial membrane potential, (**Fig. 2C**, p<0.05). ALF186 significantly attenuated mitochondrial depolarization caused by rotenone (17±4% in rotenone+vehicle vs. 55±3% in ALF186+rotenone treated, (**Fig. 2C**, p<0.05). iALF186 did not prevent mitochondrial depolarization (iALF+rotenone 15±5%).

### ALF186 Acts Independently of iNOS and nNOS

**Figure 3:**
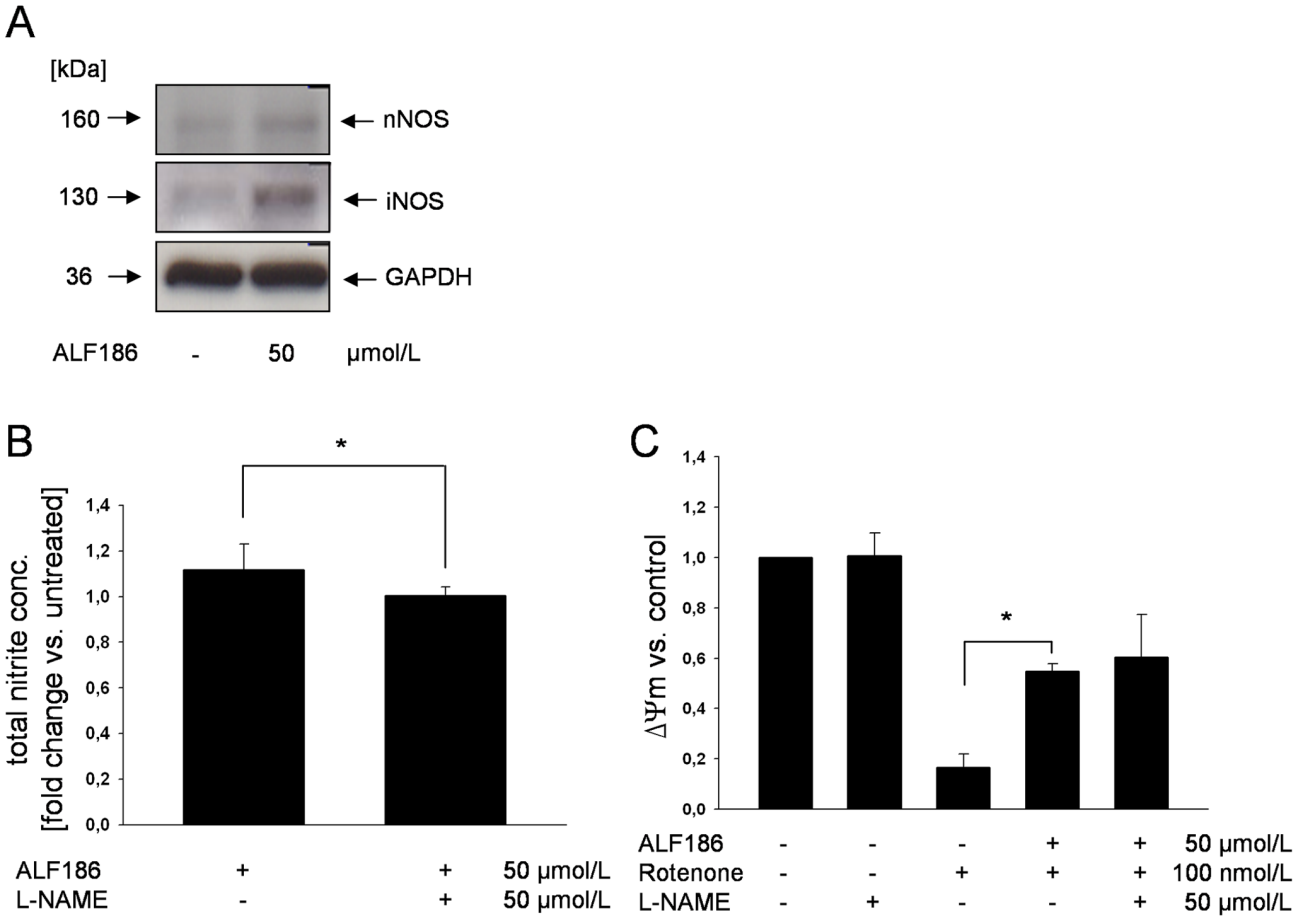
The role of the heme-containing “primary” CO-target NOS in ALF186 mediated effects. **A:** Representative western blot of nNOS and iNOS protein expression after ALF186 treatment. **B:** Quantification of nitric oxide production by colorimetric measurement of total nitrite after ALF186 incubation ±NOS-Inhibition by L-NAME (n = 6; fold change vs. untreated cells; mean±SD; * = p<0.05 ALF186 vs. L-NAME +ALF186). **C:** Flow-cytometric analysis of mitochondrial membrane potential change relative to untreated cells after inhibition of NOS (ΔΨ_m_, n = 6; mean±SD; * = p<0.05 rotenone vs. ALF186+rotenone).

To analyze the involvement of heme-containing enzymes as CO targets in mediating neuroprotection, we next investigated whether ALF186 influenced activity and expression of the NOS enzymes. ALF186 induced expression of both the inducible and neuronal (n) isoforms of NOS (**Fig. 3A**). ALF186 slightly increased nitric oxide production and this increase was abolished in the presence of the NOS-Inhibitor L-NAME (**Fig. 3B**, p<0.05). However, inhibition of NOS with L-NAME did not influence ALF186 mediated protection against rotenone (**Fig. 3C**, rotenone 17±5% of mitochondrial membrane potential vs. ALF186 50 µmol/L+rotenone 55±3% and vs. L-NAME+ALF186+rotenone 60±17%, both p<0.05).

### ALF186 Requires Reactive Oxygen Species Originating from NADPH Oxidase in SH-SY5Y Cells for Protection

**Figure 4:**
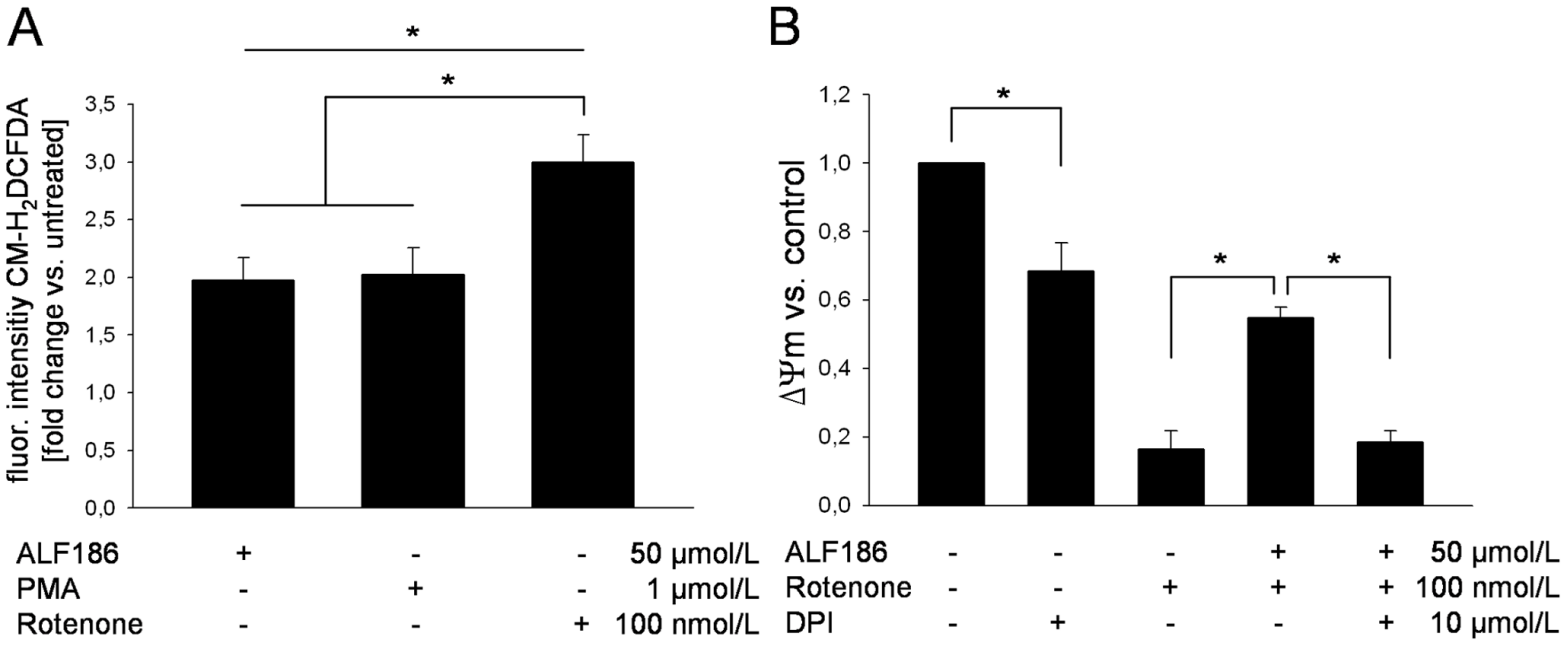
Influence of ALF186 on ROS production and role of NADPH oxidase in ALF186 mediated effects. **A:** Flow-cytometric analysis of ROS production after CM-H_2_DCFDA staining (n = 6; mean±SD; * = p<0.05 all vs. untreated; rotenone vs. ALF186 and vs. PMA). **B:** Flow-cytometric analysis of mitochondrial membrane potential change relative to untreated cells after NADPH oxidase inhibition (ΔΨ_m_, n = 6; mean±SD; * = p<0.05 DPI vs. untreated; rotenone vs. ALF186+rotenone and ALF186+rotenone vs. DPI+ALF186+rotenone).

CO has been shown to transiently increase ROS in macrophages by targeting the mitochondria. The effect of CO on ROS generation in neurons has not been tested. Given the ability of CO to protect cells against rotenone, we turned to NADPH oxidase as an alternative hemoprotein target given that it also generates ROS formation and regulates cell signaling. To determine whether ALF186 influences intracellular ROS production we incubated SH-SY5Y cells with the oxidant dye CM-H_2_DCFDA and analyzed ROS production by flow-cytometry. Incubation with ALF186 alone resulted in a significant increase in intracellular ROS production comparable to the known NADPH oxidase inducer PMA (**Fig. 4A**). Increases in ROS production by ALF186 or PMA were moderate compared to the strong induction of ROS by rotenone (**Fig. 4A**). Inhibition of NADPH oxidase with diphenylene iodonium (DPI) resulted in abolishment of ALF186 mediated protection (**Fig. 4B**). Interestingly, incubation with DPI alone resulted in a significant loss in mitochondrial membrane potential (69±8% vs. untreated, p<0.05) suggesting either that baseline activity of NADPH oxidase in generating ROS is required for survival or that DPI has other toxic effects on mitochondria.

### ALF186 Induces the Activity and Expression of sGC

**Figure 5:**
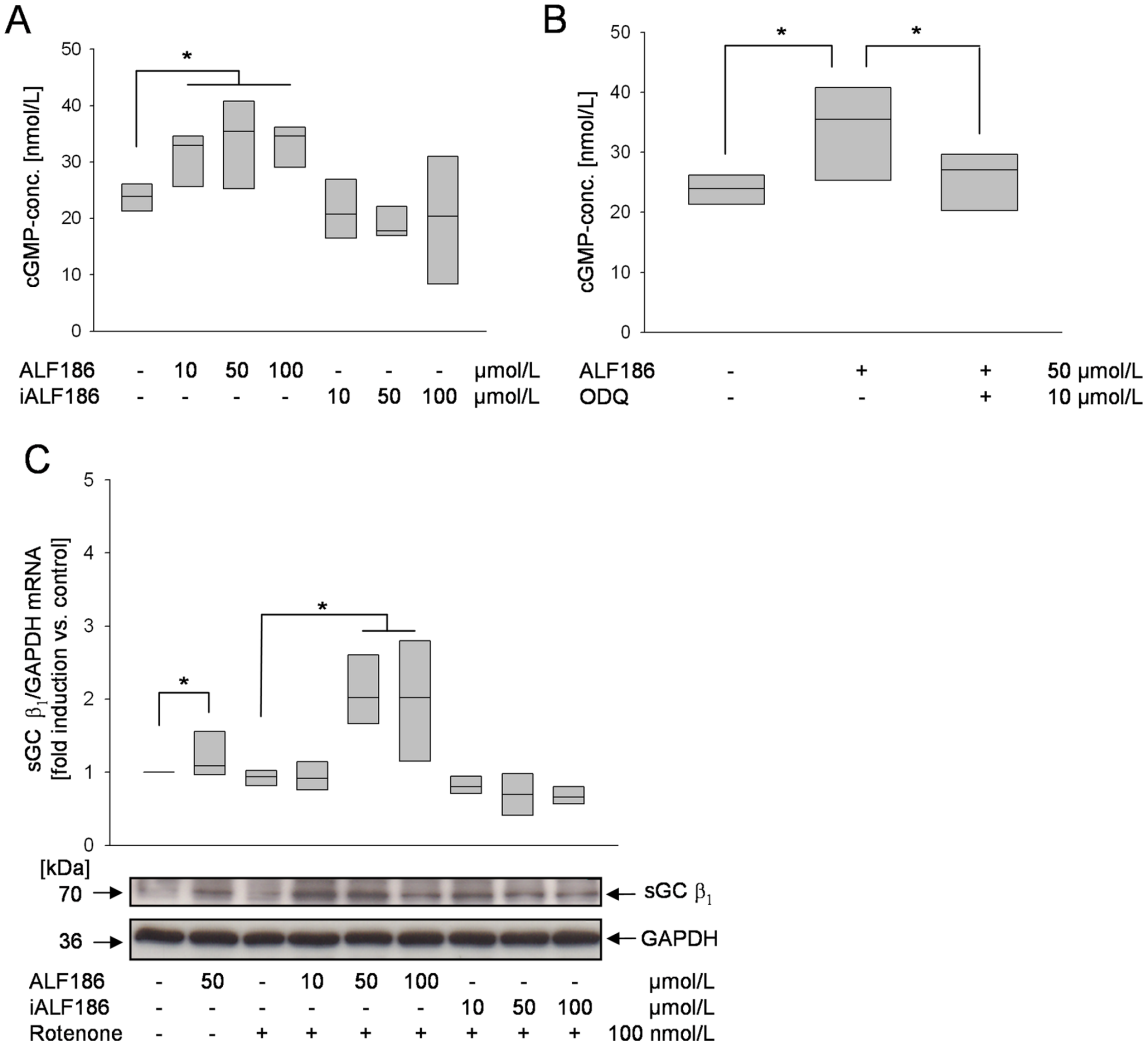
Effect of ALF186 on cellular cGMP levels and expression of sGC. **A:** ELISA analysis of cellular cGMP concentration after ALF186 treatment (n = 6; median±25^th^/75^th^ percentile; * = p<0.05 untreated vs. ALF186 10, 50, 100 µmol/L). **B:** Analysis of cellular cGMP concentration after ALF186 treatment ± sGC-Inhibition by ODQ (n = 6; median±25^th^/75^th^ percentile; * = p<0.05 untreated vs. ALF186 and ALF186 vs. ODQ +ALF186). **C:** Effect of ALF186 on sGC β_1_ subunit mRNA (top; RT-PCR analysis, n = 6; median±25^th^/75^th^ percentile; * = p<0.05 untreated vs. ALF186 50 µmol/L and rotenone vs. ALF186 50, 100 µmol/L+rotenone) and protein (bottom, representative Western Blot of sGC β_1_ protein) expression.

To further analyze the influence of ALF186 on heme-containing potential carbon monoxide targets, we next looked at soluble guanylate-cyclase (sGC), which is known to be a key target in vascular smooth muscle [15]. ALF186, but not iALF186 significantly increased the amount of cellular cGMP, the reaction product of sGC from 23±3 nmol/L in control cells to 33±5 nmol/L in the presence of 100 µmol/L ALF 186 (**Fig. 5A** **,** p<0.05). Increases in the amount of cellular cGMP by ALF186 were effectively blocked by the sGC-Inhibitor ODQ (**Fig. 5B**, p<0.05). The increase in cGMP likely reflects the ability of CO to influence sGC activity, however RT-PCR and Western blot analysis showed that the expression of the sGC β_1_ subunit was also induced by ALF186 and not by iALF186 (**Fig. 5C**).

### Inhibition of sGC Activation or the sGC Downstream Target PKG1 Abrogates ALF186 Mediated Protection

**Figure 6:**
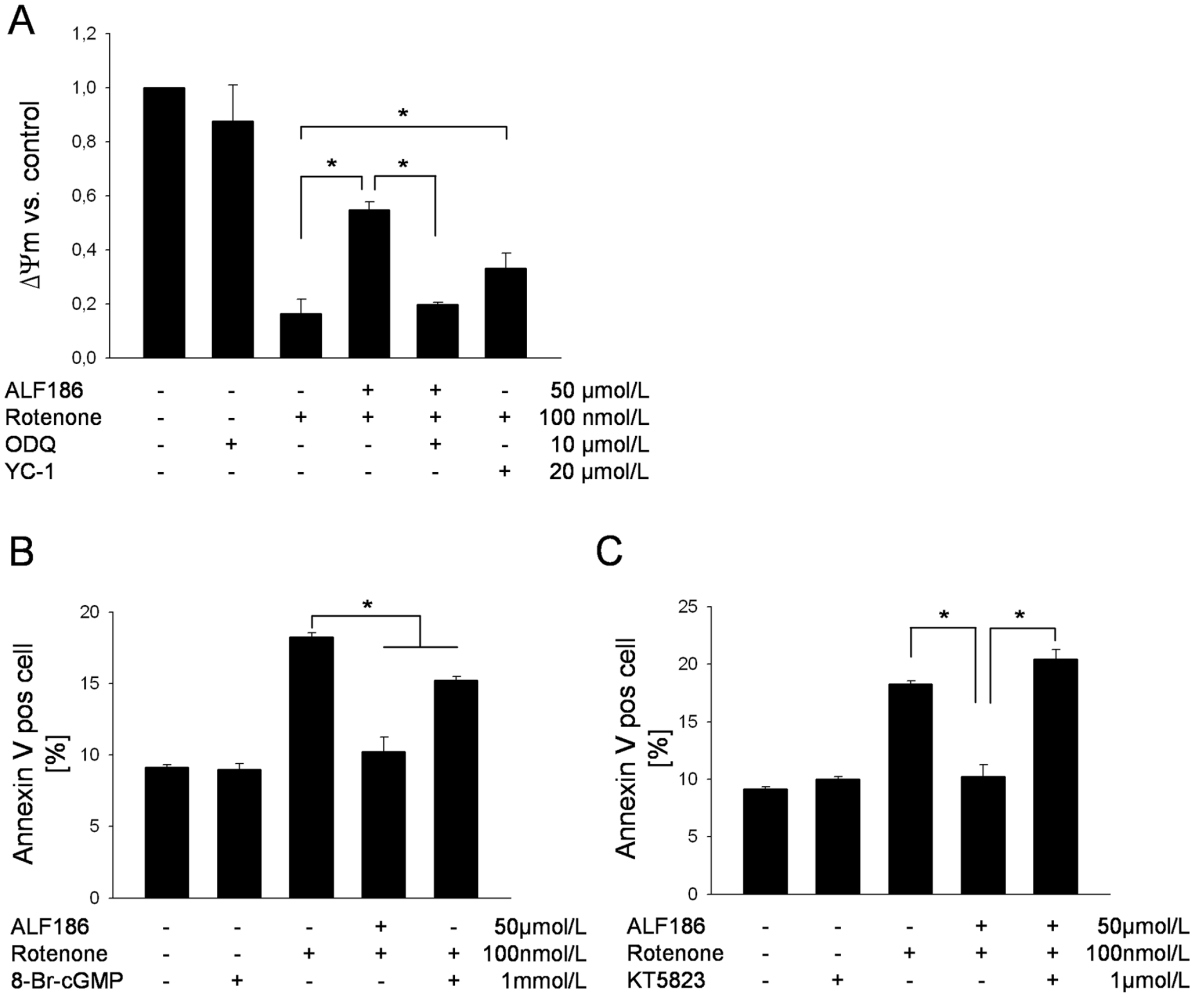
The role of sGC in ALF186 mediated effects. **A:** Flow-cytometric analysis of mitochondrial membrane potential change relative to untreated cells after inhibition or induction of sGC (ΔΨ_m_, n = 6; mean±SD; * = p<0.05 rotenone vs. ALF186+ rotenone, ALF186+rotenone vs. ODQ+ALF186+rotenone and rotenone vs. YC-1+rotenone). **B:** Flow-cytometric analysis after Annexin V staining and incubation with the cGMP-analog 8-Br-cGMP (n = 6; mean±SD; * = p<0.05 rotenone vs. ALF186+rotenone and rotenone vs. 8-Br-cGMP+rotenone). **C:** Flow-cytometric analysis after Annexin V staining and incubation with the PKG-inhibitor KT5823 (n = 6; mean±SD; * = p<0.05 rotenone vs. ALF186+rotenone and ALF186+rotenone vs. KT5823+ALF186+rotenone).

We next asked whether the effects of ALF186 required sGC activity. Inhibition of sGC activation with the selective inhibitor ODQ prior to ALF186 incubation and rotenone-treatment led to mitochondrial depolarization comparable to treatment with rotenone alone and effectively reversed the protection afforded by ALF186 (**Fig. 6A**; 17±5% rotenone vs. 55±3% ALF186+rotenone vs. 20±1% ODQ+ALF186+rotenone, p<0.05). Induction of sGC with YC-1 stabilized mitochondrial membrane potential (rotenone 17±5% of the mitochondrial membrane potential vs. YC-1+rotenone 33±6%, p<0.05), even though this effect was less pronounced than with ALF186. We next mimicked sGC activation by incubating cells with the cell-permeable cGMP-analogue 8-Br-cGMP. Indeed, 8-Br-cGMP afforded significant protection, which was not as pronounced as that observed with ALF186 treatment (**Fig. 6B**). Several downstream targets are modulated by cGMP, including PKG1. We now asked whether the cGMP-mediated protective effects were dependent on PKG1 activity. PKG1-inhibition with KT5823 abolished ALF186 mediated protection (**Fig. 6C**).

### ALF186 Protects Retinal Ganglion Cells against Ischemia Reperfusion Injury *in vivo* through sGC

In a last set of experiments we translated our *in vitro* results into an *in vivo* model of neuronal cell damage. Retinal ganglion cells (RGC) are known to be exceptionally susceptible to cell death via apoptosis when subjected to IRI. We therefore used a well-described *in vivo* model of retinal IRI [3] to investigate potential neuroprotective effects of ALF186 *in vivo*.

**Figure 7:**
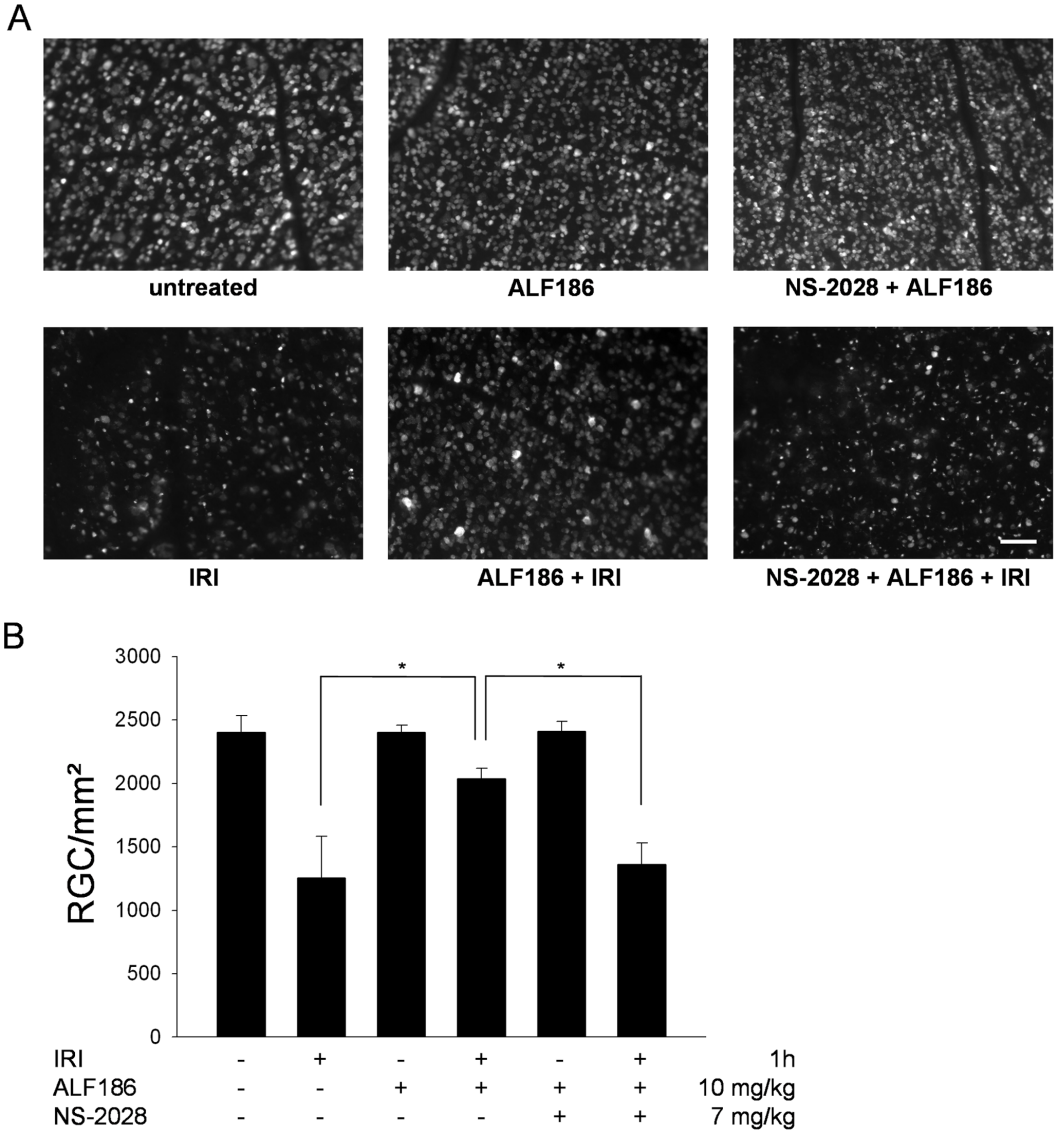
Effect of ALF186 treatment on IRI in retinal ganglion cells *in vivo*. **A:** Representative images from flat mounts with fluorogold-labeled retinal ganglion cells 7 days after IRI, ALF186 treatment and sGC inhibition with NS-2028. Scale bar 100 µm. **B:** Quantification of retinal ganglion cell density [cells/mm^2^] 7 days after IRI, ALF186 treatment and sGC inhibition *in vivo* (n = 6 per group; mean±SD; * = p<0.05 IRI vs. ALF186+IRI and ALF186+IRI vs. NS-2028+ALF186+IRI).

Density-quantification of fluorogold-labeled RGC showed that retinal tissue from animals receiving intravenous ALF186 treatment before injury had significantly higher ganglion cell densities compared to animals treated with vehicle alone (**Fig. 7A–7B** **;** IRI 1255±327 RGC/mm^2^ vs. ALF186 10 mg/kg+IRI 2036±83, p<0.05). RGC densities in corresponding control eyes showed no significant difference from each other (**Fig. 7A–7B**). *In vivo* co-treatment of animals with the sGC-inhibitor NS-2028 abolished the protective effects mediated by ALF186 (**Fig. 7A–7B**; ALF186 10 mg/kg+IRI 2036±83 RGC/mm^2^ vs. NS-2028 7 mg/kg+ALF186 10 mg/kg+IRI 1263±170, p<0.05).

**Figure 8:**
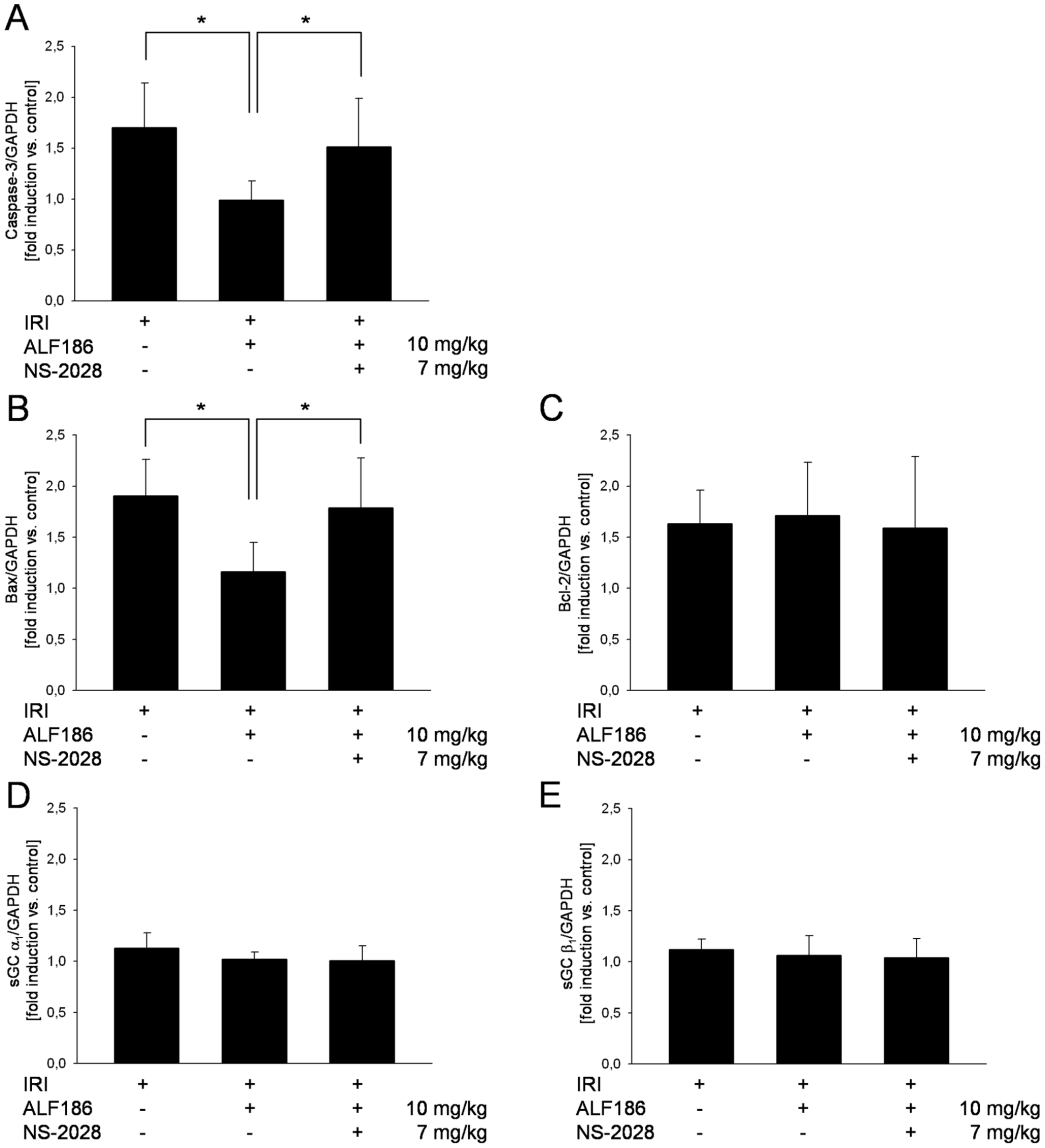
Effect of ALF186 on retinal caspase-3, Bax, Bcl-2 and sGC-subunit gene expression analyzed by RT-PCR. Retinal expression of caspase-3 (**A**; n = 6; mean±SD.; * = p<0.05 IRI vs. ALF186+IRI and ALF186+IRI vs. NS-2028+ALF186+IRI), Bax (**B**; n = 6; mean±SD; * = p<0.05 IRI vs. ALF186+IRI and ALF186+IRI vs. NS-2028+ALF186+IRI), Bcl-2 (**C**; n = 6; mean±SD), sGC α_1_ subunit (**D**; n = 6; mean±SD) and sGC β_1_ subunit (**E**; n = 6; mean±SD) mRNA expression in ischemic retinal tissue in relation to the corresponding non-ischemic retinae analyzed by RT-PCR.

To confirm these *in vivo* results we analyzed the retinal gene expression of pro-apoptotic (Caspase-3, Bax) and anti-apoptotic (Bcl-2) genes using RT-PCR. Expression of Caspase-3 and Bax was significantly suppressed by ALF186 treatment, whereas sGC-inhibition abolished this anti-apoptotic effect (**Fig. 8A–8B**, Bax: ALF186 10 mg/kg+IRI 1.1±0.3 fold induction vs. NS-2028 7 mg/kg+ALF186 10 mg/kg+IRI 1.8±0.4, p<0.05). Expression of Bcl-2 was not altered, by any treatment (**Fig. 8C**). In contrast to *in vitro* gene expression analyses of the sGC subunits α_1_ and β_1_ expression (**Fig. 8B**), *in vivo* was not altered by ALF186 (**Fig. 8D–E**).

## Discussion

The main findings can be summarized as follows: (1) Pre-treatment with CORM ALF186 attenuates rotenone-induced apoptosis in SY5Y neuroblastoma cells. (2) ALF186 preconditioning influences several heme-containing cellular “primary” targets: (a) NO-Synthase, (b) NADPH-oxidase-derived ROS and (c) sGC. (3) ALF186 protects RGC from IRI through inhibition of apoptosis *in vivo* that likely involves a similar pathway since sGC inhibition abolishes the protective effects. We therefore hypothesized that CO arising from ALF186 mediated a series of interrelated cascades involving a cGMP/NOS/ROS pathway since inhibition of these pathways at least *in vitro* abolishes the protective effects. Protection against apoptosis required cGMP and ROS generation, but not NOS. We acknowledge that the link between these signaling molecules requires further study, but we can conclude that preconditioning with CORM ALF186 protects neuronal cells *in vitro* and *in vivo* from apoptosis and IRI and that these effects were abrogated when we used the inactive, CO-free ALF186.

Carbon monoxide, a dose-dependent therapeutic gas has profound effects in inhibiting systemic inflammation and apoptosis in numerous *in vitro* and *in vivo* models when given either as a pre- or postconditioning agent resulting in organoprotective actions [10], [21]–[28]. CO can also exert protective effects on neuronal cells *in vitro* [2], [29] and *in vivo* in small and large animal models [3], [30], [31]. The potential human application of CO gas is challenging to implement not only for technical reasons, which have been addressed [10], but also for intrinsic flaws including lack of tissue specificity and at present time, hospital-only administration [32]. For these reasons, it is crucial to find alternative routes for the application of CO. CORMs have emerged as an excellent alternative for delivering CO and have the potential for tissue specificity. ALF186 does not carry such attributes, yet does clearly demonstrate the feasibility of parenteral delivery. ALF186 is one of a family of CORMs that are orally active leading to similar elevations in COHb as when given intraperitoneally or intravenously (data not shown) similar to other CORMs [4], [13], [17], [33]–[39]. Therefore CORMs represent a safe alternative for potential clinical application of CO [32]. Unlike CO gas, which has very simple pharmacokinetics, the CORMs carry more complicated pharmacologic challenges that need to be assessed.

To date studies on the effects of CORM-3 on neuronal cells and neuroinflammation have provided inconsistent results [4], [5]. With the advent of the molybdenum-containing, water-soluble CORMs such as ALF186, CO is released upon contact with oxygen. It has little sensitivity to light and thus features predictable and favorable CO releasing kinetics with a half life of approximately 15 minutes in aqueous solvents and media *in vitro* [6] and fast, bolus-like CO releasing kinetics *in vivo* [6], the latter of which is likely due to the interaction with hemoglobin.

The *in vitro* model of rotenone-induced apoptosis in SY5Y neuroblastoma cells is a well-established model of caspase-dependent neuronal apoptosis [40], [41]. It has been extensively used to study potential neuroprotective effects of endogenous mediators like prostaglandins [42] or chondroitin sulfate [43] and drugs like valproic acid [44], hydrogen sulfide [45], tranexamic acid [46], histamine receptor antagonists [47] or deferoxamine [48]. Consistent with these previous studies, we found increased Annexin V positivity, caspase-3 cleavage and mitochondrial depolarization after rotenone-treatment. Pre-treatment with ALF186 attenuated apoptosis, which was evidenced by decreased Annexin V positivity, inhibition in caspase-3 cleavage and stabilization of mitochondrial membrane potential all of which were not observed in control experiments with the CO-free inactive ALF186 (iALF186).

Since CO avidly binds to heme groups of cellular structures such as NOS, NADPH oxidase or sGC, it is tempting to assume that the effects of CO are mediated through alteration of such “primary” CO targets. For example, CORM-2 and CORM-3 exhibit anti-inflammatory effects through reduced NO−/nitrite-production and iNOS expression [11], [35], [49]. In contrast to these results, we found that while ALF186 mediated an increase in iNOS and nNOS protein expression in SY5Y cells, it was not involved in protection.

CORM-2 exhibited anti-proliferative effects in smooth muscle cells by blocking NADPH oxidase and the cellular redox system [12]. In contrast, NADPH oxidase derived ROS can promote cell survival through increased endogenous CO production in endothelial cells [50]. Others have found that CO gas mediates increased mitochondrial-dependent ROS production in macrophages [51], [52]. In neuronal SY5Y cells, we also observed an ALF186-mediated increase in ROS production, which we determined to be most likely arising from NADPH oxidase activity as protection was inhibited by DPI, a selective NADPH oxidase inhibitor. Perhaps different modes of CO delivery (gas vs. CORM) can influence the ROS-generating systems of the cell or perhaps speaks to differences in cell types, neurons vs. macrophages.

Vasorelaxing properties of CORM-3, CORM-A1 and ALF186 have been associated with the induction of the hemoprotein sGC and a moderate, but significant increase in cGMP production, activation of PKG1 and downstream activation of calcium-sensitive potassium channels [9], [13], [15], [53]. Moreover, CO increases retinal and choroidal blood flow in human eyes [54]. However, it is not known whether CORM mediated sGC induction also modulates neuroprotection. sGC is a heterodimer, consisting of an α (1 or 2) and heme-containing β (1 or 2) subunit. Heterodimerization of the enzyme’s α 1 and β 1 subunits is essentially necessary for the enzyme’s full activity [55], [56]. There is growing evidence that activation of the sGC-cGMP pathway may also exert cytoprotective and anti-apoptotic effects in neuronal cells [16]. However, no study has investigated possible CORM-mediated protective effects in neuronal cells through this pathway. Protective effects by activation of sGC-cGMP signal transduction also seem to be mediated through PKG1 activation [16], but little is known about the downstream targets of PKG1 that mediate these effects.

The findings of the *in vivo* study are in accordance with our previous findings in the same experimental model of retinal injury, where administration of inhaled CO gas before ischemia was anti-inflammatory, anti-apoptotic and exerted cytoprotection of RGC [3]. The degree of neuroprotection was comparable between both the application of CO gas and ALF186. We delineate here that ALF186 mediates its neuroprotective effects *in vivo* through a sGC-dependent pathway just as the *in vitro* experiments have demonstrated. The neuronal cell type and injury model kinetics that we used *in vitro* and *in vivo* differed from one another. However, since RGC undergo apoptosis after IRI [57], [58] similarly to SH-SY5Y cells do in response to rotenone, both models can be used to assess apoptosis-related neuronal cell death and CO-mediated neuroprotection.

Taken together, this study demonstrates that preconditioning with the novel CORM ALF186 protects neuronal SY5Y and retinal ganglion cells against ischemic insult in part via a sGC dependent pathway. CO released from CORMs may therefore be a treatment option for acute ischemic insults to the retina and the brain by protecting neurons.
