# Correction: Carbon Monoxide Abrogates Ischemic Insult to Neuronal Cells via the Soluble Guanylate Cyclase-cGMP Pathway

**DOI:** 10.1371/annotation/f047d50f-761b-4c99-9e13-d2526fde71b7

**Published:** 2013-11-01

**Authors:** Nils Schallner, Carlos C. Romão, Julia Biermann, Wolf A. Lagrèze, Leo E. Otterbein, Hartmut Buerkle, Torsten Loop, Ulrich Goebel

The following information was missing from the Funding section:

"The article processing charge was funded by the German Research Foundation (DFG) and the Albert Ludwigs University Freiburg in the funding programme "Open Access Publishing".

There is an error in the Competing Interests section. The correct Competing Interests statement is:

"The authors have read the journal's policy and have the following conflicts: Author Carlos C. Romão is a co-founder, administrator and scientific officer of the company Alfama Lda. where he has equity interests. CC Romão is an inventor in Patent-No.: US 2011/0038955 A1 2011 where ALF186 exemplifies the protection of the stomach against NSAID generated ulcers by the administration of CORMs, and is therefore irrelevant for the matter of the present article. The drug profile of ALF186 is published in detail in: Dalton Transactions DOI: 10.1039/c2dt32174b. Author Leo E. Otterbein as a former collaborator of Alfama Lda. no longer has any professional relationship or equity interests in the company. These conflicts of interest do not alter the authors' adherence to all the PLOS ONE policies on sharing data and materials. The remaining authors declare no competing financial interests.”

